# Risk factors for complex posttraumatic stress disorder in UK police

**DOI:** 10.1093/occmed/kqab114

**Published:** 2021-08-20

**Authors:** C Steel, N Tehrani, G Lewis, J Billings

**Affiliations:** 1 Division of Psychiatry, University College London, London, UK; 2 Noreen Tehrani Associates, UK

**Keywords:** Complex posttraumatic stress disorder, occupational health, police officers, risk factors

## Abstract

**Background:**

Police officers are frequently exposed to distressing and dangerous situations, increasing their risk of posttraumatic stress disorder (PTSD) and complex PTSD (C-PTSD). Research examining C-PTSD in police officers is sparse, particularly examination of the occupational risk factors for trauma symptoms.

**Aims:**

This study aimed to examine the prevalence and risk factors for PTSD and C-PTSD in UK police officers.

**Methods:**

A cross-sectional study was conducted using psychological health surveillance data from the UK National Police Wellbeing Service. Police officers were either from high-risk areas of work or had been referred for screening by occupational health practitioners regarding psychological distress. The primary outcome for this study was a positive screening of either PTSD or C-PTSD, measured using the International Trauma Questionnaire. A range of occupational, clinical and lifestyle factors was examined to establish their role as potential risk factors for PTSD and C-PTSD.

**Results:**

In total, 2444 UK police officers were included, with 89% from high-risk areas of work. A prevalence of 3% for PTSD and 2% for C-PTSD was found in police officers from high-risk areas of work. Higher work stress and lower manager support were found to increase the odds of C-PTSD but not PTSD. Higher personal trauma history increased the risk for PTSD and C-PTSD equally.

**Conclusions:**

Work-related occupational factors increased the odds of PTSD and C-PTSD in police officers, which could be important risk factors for trauma symptoms within police officers. Efforts should be made to improve the working environment of police officers to help improve their psychological well-being.

**Key learning points**

**What is already known about this subject:**

- Frequent exposure to traumatic events at work put police officers at an increased risk of complex posttraumatic stress disorder [11].
- Some occupational factors have already been shown as potential risk factors for complex posttraumatic stress disorder in police officers, including exposure to sexual harassment and humiliating behaviours at work, lower rank and higher tenure [11].

**What this study adds:**

- This study demonstrated that additional occupational factors, especially high work stress and lower perceived manager support, are more strongly associated with complex posttraumatic stress disorder symptoms than self-reported level of trauma exposure.
- This study gives support to the possible presence of occupational trauma in police officers, which presents differently from posttraumatic stress disorder and complex posttraumatic stress disorder.

**What impact this may have on practice or policy:**

- Police services need to ensure a sound working environment, for example sufficient manager support, to minimize the impact of trauma exposure.
- This could be facilitated through developing and implementing well-being programmes in police services to cultivate resilience among individuals and teams and greater awareness of mental health issues among managers.

## Introduction

Due to frequent exposure to distressing and dangerous situations, police officers are at an increased risk of posttraumatic stress disorder (PTSD). A pooled prevalence estimate revealed that approximately 14% of police officers worldwide meet diagnostic threshold for PTSD [1]. However, less is known regarding complex PTSD (C-PTSD) in police officers. C-PTSD is defined by the same diagnostic criteria as PTSD, with the additional criteria of disturbances in self-organization, including affect dysregulation, negative self-concept and interpersonal disturbances. Given the risk of PTSD, further investigation of both trauma-related disorders in police populations is necessary.

Repetitive or prolonged exposure to traumatic events is recognized as a significant risk factor for C-PTSD [2]. Cross-sectional analysis has demonstrated that a higher accumulation of adverse childhood experiences (ACEs) is strongly associated with more severe C-PTSD symptoms [3]. Moreover, latent analysis has established that C-PTSD is strongly associated with prolonged and severe adulthood trauma [4]. Given the repeated exposure to traumatic events in policing, and reports that 20% of police recruits have experienced ACEs [5], this combination of personal and occupational traumatic exposure in police populations might increase the incidence of C-PTSD.

As well as trauma history, a cross-sectional study has found lower perceived social support as uniquely associated with C-PTSD over PTSD [6]. This may be relevant in police populations where high workload and unsociable working hours are common. Moreover, a cross-sectional study of the UK population established that alcohol abuse doubled the odds of C-PTSD [7], and in a study of military veterans those with C-PTSD reported poorer sleep quality compared with those with PTSD [8]. With 5% of police officers reporting increased alcohol consumption following a work trauma [9], and meta-analyses demonstrating that 51% of police officers have below average sleep [10], alcohol intake and sleep quality may be crucial risk factors for C-PTSD in police officers. Finally, cross-sectional studies reveal that individuals with C-PTSD have increased anxiety and depression symptoms than those with PTSD [7]. Thus, individual-level clinical factors, interpersonal and lifestyle characteristics should be considered when examining C-PTSD in police populations.

Currently, only one study has examined C-PTSD in police personnel. In a recent survey of over 16 000 UK police officers, approximately 13% met the diagnostic threshold for C-PTSD [11]. Factors increasing the risk of C-PTSD included exposure to sexual harassment and humiliating behaviours at work, lower rank and higher tenure. However, the police personnel in this study were seeking support for psychological difficulties, therefore a potentially biased sample. Moreover, additional occupational factors may have been involved requiring further investigation.

We hypothesize that compassion fatigue, burnout and compassion satisfaction may contribute to C-PTSD risk among police officers. Moreover, specific policing roles may be relevant as different types of trauma exposure may impact the risk of C-PTSD [12]. Finally, greater colleague support and sense of coherence in work are associated with high posttraumatic growth following a recent trauma [13]. To obtain a more in-depth understanding of C-PTSD in police populations, it is necessary to examine the role of these additional occupational factors.

In this study, we aimed to examine the prevalence of PTSD and C-PTSD within a sample of high-risk UK police officers, and the role of occupational, clinical and lifestyle as risk factors for PTSD and C-PTSD.

## Methods

We used cross-sectional data from the Noreen Tehrani Associates Psychological Screening (NTAPS) programme as part of the National Police Wellbeing Service. The data are gathered under Health and Safety Legislation for health surveillance purposes. Data from January to December 2019 were used. The primary outcome was a positive screening for PTSD or C-PTSD. Exposures included occupational, clinical and lifestyle factors.

Participants were UK police officers in forces participating in the NTAPS programme. Participants were either in a high-risk role undertaking routine screening or were referred for screening by occupational health as presenting with psychological distress. The National Police Wellbeing Service identifies high-risk police roles using College of Policing definitions. Every police officer in a high-risk role or referred was invited for screening. According to service records, approximately 80% of police officers invited will undertake the screening. Both routine and referral screenings were included to obtain an accurate representation of all police officers undertaking an initial screening.

Following screening, officers with clinically significant scores for psychological distress were referred for an assessment by an occupational health practitioner. These officers were provided with well-being guidance (for example, lifestyle and coping workbooks or workshops) or were referred for trauma therapy.

Individual data from the NTAPS service is protected by a Privacy Policy and Notice governed by the law of England and Wales. Police forces provided e-mail addresses of the officers identified to be screened for NTAPS to send the questionnaires. All police officers received an information booklet explaining the screening, contact details, the NTAPS privacy policy and a consent statement to electronically sign (see Supplementary Material, available as Supplementary data at *Occupational Medicine* Online). Police officers were made aware that their anonymous data may be used for research and provided consent for this purpose. The NTAPS service is responsible for the database holding the screening data. All police officers who undertook the screening were included on the database regardless of their scores.

All police officers were given identical screening questionnaires. Screening outcome for PTSD and C-PTSD was assessed using the International Trauma Questionnaire (ITQ) [14], a self-report diagnostic measure of ICD-11 PTSD and C-PTSD. Screening outcome is either positive or negative for PTSD or C-PTSD. The construct and discriminant validity of the ITQ is well established in trauma-exposed patients [15].

ACEs were assessed through a self-report questionnaire taken from a validated survey instrument demonstrated to have good test–retest reliability [16]. Questions regarding other personal traumas were taken from a trauma history questionnaire developed by the Post Office Employee Support Service [17]. The questions included a list of possible recent events, adult events and addictive behaviours. Higher scores indicate more personal traumas.

Anxiety and depression were measured through the Goldberg Anxiety/Depression Scale [18], a self-report questionnaire. Total scores range from 0 to 9 for each subscale. Higher scores indicate more severe symptoms. The scale has good predictive validity [18], internal consistency and construct validity within an occupational setting [19].

Factors relating to professional quality of life, including ‘compassion fatigue’, ‘compassion satisfaction’ and ‘burnout’ were assessed using the Professional Quality of Life (ProQOL) [20] self-report measure. Higher scores on ‘burnout’ and ‘compassion fatigue’, and lower scores on ‘compassion satisfaction’ indicate negative outcomes. The ProQOL has been demonstrated to have good reliability and construct validity [20].

Personal resilience was measured through the self-report Sense of Coherence (SoC) scale [21]. There are three subscales: ‘meaningfulness’, ‘comprehensibility’ and ‘manageability’. Lower subscale scores indicate lower personal resilience. The SoC is reliable, valid and cross-culturally applicable, with good predictive validity of future health outcomes [22].

A lifestyle questionnaire (see Supplementary Material, available as Supplementary data at *Occupational Medicine* Online) was developed by NTAPS based on well-being guidance from the National Health Service. Each item refers to a different lifestyle factor, scored as either good, average or poor. Only alcohol intake, socialization and sleep were included in this study.

The Emotional Literacy Questionnaire (ELQ) [23] was used to assess emotional resilience and somatic sensitivity to traumatic events. The ELQ is a self-report questionnaire consisting of seven subscales. Subscale scores range from 0 to 6. Low scores on ‘dissociation’ and ‘sensory awareness’ and high scores on all other subscales indicate problematic emotional resilience.

Questions regarding employment background (see Supplementary Material, available as Supplementary data at *Occupational Medicine* Online) were derived by NTAPS to assess the police officers’ perspective on factors that might impact their work performance. The individual items of this questionnaire have been demonstrated to have criterion validity with established questionnaires. Higher scores on ‘exposure to work trauma’, ‘intentions to leave’ and ‘work stress’, and lower scores on ‘health beliefs’, ‘workability’ and ‘manager support’ indicate lower performance in these areas. Tenure was provided from 10 options (see Table 2). Police roles, in line with College of Policing classifications, were as follows: ‘Investigations’ (for example, public protection, counter terrorism and forensics); ‘Community Policing’ (for example, response officers, offender management and safeguarding); ‘Intelligence’ (for example, cyber-crime, covert officers and surveillance); ‘Operational Support’ (for example, firearms officers, family liaison and negotiators); and ‘Unknown’ (police who chose not to disclose their work area).

Only police officers routinely screened were included in the prevalence estimates of PTSD and C-PTSD to reduce the risk of bias. Participants who were referred for screening were removed from prevalence estimates. To assess risk factors for PTSD and C-PTSD, both participants routinely screened and referred for screening were included. Univariable logistic regression analyses were conducted to assess the association between each exposure and PTSD and C-PTSD screening outcomes. Multivariable logistic regression models were used to assess the impact of age, gender, tenure and work group on these associations. Univariable logistic regression analyses were used to examine whether any variables were associated with missing data (see S1 and S2 in the Supplementary Material [available as Supplementary data at *Occupational Medicine* Online] for full details). The *P* values are reported in terms of the strength of statistical evidence using guidance from a medical statistics manual [24]. We used Stata, version 15 [25] for analyses.

## Results

In total, 2444 police officers completed screening of which 2171 (89%) were part of routine screening and 273 (11%) were referred. The mean age of the whole sample (*n* = 2444) was 39.5 years (SD = 9.5), with 1288 (53%) males. Characteristics for the whole sample can be found in Tables 1 and 2.

**Table 1.** Demographic, clinical and lifestyle characteristics of the whole sample

	Total police officers (*n* = 2444)
Demographic, clinical and lifestyle characteristics	Mean (SD)^a^ or *n* (%)
Age (years)	39.5 (9.5)
Gender (male)	1288 (53%)
Screening type (routine)	2171 (89%)
Positive screening for PTSD (ITQ)	98 (4%)
Positive screening for C-PTSD (ITQ)	165 (7%)
Anxiety (Goldberg)	3 (1–6)
Depression (Goldberg)	1 (0–4)
Personal history (*n*)	
Adverse childhood events	0 (0–1)
Adverse adult events	1 (0–2)
Recent events	0 (0–1)
Addictive behaviours	0 (0–0)
Total	2 (1–4)
Emotional awareness (ELQ)	
Dissociation	4.1 (1.9)
Physical sensitivity	1.3 (1.5)
Emotional sensitivity	1.7 (1.6)
Sensory awareness	4.2 (1.8)
Empathy	2.9 (1.7)
Interpersonal sensitivity	4.5 (1.9)
Emotional resilience	4.8 (2.0)
Alcohol (units per week)	
≤7	1892 (77%)
8–14	361 (15%)
≥15	191 (8%)
Socializing outside of work (*n* per week)	
≥3 times	301 (12%)
1–2 times	1616 (66%)
0	527 (22%)
Sleep (hours per night)	
≥7	1516 (62%)
5–6	829 (34%)
<5	99 (4%)

*n* = total number. Goldberg Anxiety/Depression Scale is an 18-item inventory measuring symptoms of anxiety and depression with possible subscale scores ranging from 0 to 9. Emotional awareness is assessed using Emotional Literacy Questionnaire, a 42-item scale measuring how someone recognizes, translates and responds to their emotions with possible subscale scores ranging from 0 to 6. ITQ is an 18-item measure assessing symptoms of C-PTSD and PTSD with scores on each subscale ranging from 0 to 36. The amount and percentage of missing data are as follows: age = 1 (<1% of the total data).

^a^Mean (SD) or median and interquartile range unless otherwise specified.

**Table 2.** Occupational characteristics of the whole sample

	Total police officers (*n* = 2444)
Occupational characteristics	Mean (SD) or *n* (%)
Professional quality of life (ProQOL)	
Compassion satisfaction	36.5 (9.2)
Burnout	25.2 (5.7)
Compassion fatigue	11.1 (9.9)
Sense of coherence (SoC)	
Meaningfulness	22.5 (5.3)
Comprehensibility	26.3 (6.5)
Manageability	20.3 (5.8)
Area of work	
Investigations	1245 (51%)
Community policing	285 (12%)
Intelligence	97 (4%)
Operational support	401 (16%)
Unknown	416 (17%)
Tenure	
Recruitment/pre-deployment	285 (12%)
0–6 months	352 (14%)
7–12 months	244 (10%)
13–18 months	209 (9%)
19–24 months	153 (6%)
2–3 years	228 (9%)
3–4 years	164 (7%)
4–5 years	135 (6%)
5–6 years	87 (4%)
>6 years	587 (24%)
Perceived exposure to traumatic material at work	
None/low	347 (18%)
Moderate	845 (43%)
High	782 (40%)
Intentions to leave role	
None	675 (34%)
Low	539 (27%)
Moderate	460 (23%)
High	300 (15%)
Health beliefs	
Poor/fair	389 (20%)
Good	707 (36%)
Excellent	878 (44%)
Workability	
Poor/fair	511 (26%)
Good	608 (31%)
Excellent	826 (42%)
Job stress	
None/mild	501 (25%)
Moderate	909 (46%)
High	564 (29%)
Perceived manager support	
Poor/fair	171 (9%)
Good	391 (20%)
Very good	827 (42%)
Excellent	585 (30%)

*n* = total number. ProQOL is a 30-item measure of the positive and negative consequences of working with others who have experienced stressful events, with possible subscale scores ranging from 10 to 50. SoC scale is a 13-item measure of personal resilience with scores ranging from 13 to 91. The amount and percentage of missing data are as follows: trauma at work = 470 (19%); intentions to leave = 470 (19%); health beliefs = 470 (19%); workability = 499 (20%); job stress = 470 (19%); and manager support = 470 (19%).

Of the 2171 police officers routinely screened as part of high-risk areas of work, 61 (3%, confidence interval [CI] 2.16–3.59) screened positively for PTSD, and 54 (2%, CI 1.87–3.23) for C-PTSD (Table 3).

**Table 3.** Percentage of positive screenings for PTSD and C-PTSD by screening type

	Positive screenings for the whole sample (*n* = 2444)		Positive screenings from police officers routinely screened (*n* = 2171)		Positive screenings from police officers referred for screening (*n* = 273)	
	*n* (%)	95% CI	*n* (%)	95% CI	*n* (%)	95% CI
PTSD (ITQ)	98 (4)	3.27–4.87	61 (3)	2.16–3.59	37 (14)	9.72–18.19
C-PTSD (ITQ)	165 (7)	5.79–7.82	54 (2)	1.87–3.23	111 (41)	34.78–46.74

*n* = total number. ITQ is an 18-item measure assessing symptoms of C-PTSD and PTSD with scores on each subscale ranging from 0 to 36.

All adjusted and unadjusted analyses for the associations between occupational factors and positive screenings for PTSD and C-PTSD can be found in Table S3 (available as Supplementary data at *Occupational Medicine* Online). After adjusting for age, gender, tenure and work group, there was weak evidence that the odds of PTSD were 68% lower in police officers working in Operational Support (odds ratio [OR] = 0.32, CI 0.13–0.82, *P* < 0.05) relative to Investigations officers, and strong evidence that the odds of C-PTSD were 89% lower (OR = 0.11, CI 0.03–0.44, *P* < 0.01) relative to Investigations officers. There was very strong evidence that the odds of PTSD were 2.88 times higher for police officers in Unknown roles (CI 1.80–4.60, *P* < 0.001) relative to Investigations officers, and the odds of C-PTSD were 8.67 times higher for police officers in Unknown roles (CI 5.91–2.72, *P* < 0.001) relative to Investigations officers.

There was very strong evidence that for every 1-point increase in compassion satisfaction score (ProQOL), the odds of C-PTSD decreased by 8% (OR = 0.90, CI 0.91–0.94, *P* < 0.001). There was very strong evidence that for every 1-point increase in burnout score (ProQOL), the odds of C-PTSD increased by 1.06 times (CI 1.03–1.10, *P* < 0.001). There was no evidence that compassion satisfaction and burnout scores changed the odds for PTSD.

There was very strong evidence that the odds of C-PTSD were 3.91 times higher for police officers with high perceived work stress (CI 2.24–6.84, *P* < 0.001) relative to those with no or mild perceived work stress. There was no evidence that level of perceived work stress changed the odds of PTSD.

There was very strong evidence that perceived manager support lowered the odds of C-PTSD, but no evidence that the odds changed for PTSD. The odds of C-PTSD were 77% lower in police officers reporting excellent perceived manager support (OR = 0.23, CI 0.13–0.40, *P* < 0.001) relative to those reporting poor or fair perceived manager support.

All adjusted and unadjusted analyses for the associations between clinical and lifestyle factors and positive screenings for PTSD and C-PTSD can be found in Table S4 (available as Supplementary data at *Occupational Medicine* Online). After adjustment for age, gender, tenure and area of work, there was very strong evidence that for every 1-point increase in anxiety score, the odds of PTSD increased by 1.39 times (CI 1.27–1.52, *P* < 0.001), and the odds of C-PTSD were greater, increasing by 1.88 times (CI 1.68–2.09, *P* < 0.001).

There was very strong evidence that for every 1-point increase in depression score, the odds of PTSD increased by 1.29 times (CI 1.19–1.40, *P* < 0.001), and the odds of C-PTSD were greater, increasing by 2.08 times (CI 1.86–2.33, *P* < 0.001).

There was very strong evidence that for police officers getting <5 h of sleep per night, the odds of C-PTSD was 13.40 times higher (CI 7.52–23.89, *P* < 0.001) relative to those getting ≥7 h of sleep per night, but no evidence that <5 h of sleep per night changed the odds for PTSD.

## Discussion

In this study, 3% of police officers from high-risk roles screened positively for PTSD, and 2% for C-PTSD. Higher levels of perceived work stress increased the risk of C-PTSD, and good levels of perceived manager support lowered the risk of C-PTSD. Higher levels of compassion satisfaction and burnout decreased and increased the risk of C-PTSD, respectively. The risk of anxiety and depression was greater for C-PTSD than PTSD. Poorer sleep quality increased the risk of C-PTSD but not PTSD. Finally, higher accumulative personal trauma history increased the risk equally for PTSD and C-PTSD.

As the study is cross-sectional, the direction of the identified risk factors cannot be established. Furthermore, several demographic variables were not measured, for example psychiatric history and socioeconomic characteristics. These variables could not be controlled for, meaning that their impact on the risk of PTSD and C-PTSD in this sample is unknown. There was also a risk of attrition bias, as police officers who retired prematurely due to trauma symptoms would not have been included. Thus, the prevalence estimates and risk factors must be interpreted with caution.

Only police officers within high-risk job groups or those presenting with psychological disturbances were included, limiting the generalizability of the prevalence estimates. Nevertheless, police officers in high-risk groups are those at a heightened risk of trauma symptoms, warranting urgent clinical attention.

Multiple testing was not adjusted for as formal adjustments, such as Bonferroni, are overly conservative in large samples and increase the risk of Type II error [26]. Instead, we followed an approach more frequently used in epidemiology whereby the results are interpreted using the effect size and precision. Furthermore, univariable analysis does not consider the contribution of other exposures. Although multivariable modelling would resolve this, their use in cross-sectional studies is misleading due to a lack of temporality [27]. Thus, risk factors were assessed individually for a more valid interpretation.

These findings indicate that police officers working in high-risk roles are at risk of developing PTSD and C-PTSD. However, the prevalence of C-PTSD in this study was significantly lower than previous research [11]. As the present study included police officers regardless of their psychological well-being, the prevalence estimates are likely to be lower than previous research using police officers presenting with psychological disturbances [11]. Health surveillance programmes could also increase the risk of socially desirable responding, as employees may have concerns over the implications of their responses. Thus, the use of health surveillance data in non-treatment seeking police officers may have lowered C-PTSD prevalence in the present study.

The association between perceived manager support and work stress with C-PTSD is consistent with evidence that lower interpersonal support may be a risk factor for C-PTSD [4,6]. Furthermore, higher levels of compassion satisfaction (the personal sense of accomplishment derived from an occupation [20]) decreased the risk of C-PTSD. As compassion satisfaction is often associated with the pleasure in helping others and positive feelings towards colleagues [20], interpersonal experiences both with the public and colleagues may impact the risk of developing C-PTSD in police officers. Hence, compassion satisfaction may act as a protective factor against interpersonal disturbances observed in C-PTSD.

Compassion fatigue (the mental and physical exhaustion of helping those with traumatic stress [20]) increased the risk of C-PTSD. In a cross-sectional study of police officers working with victims of rape, high scores of compassion fatigue were associated with clinically significant secondary traumatic stress (trauma symptoms resulting from exposure to the traumatic experiences of others) [28]. Together with the present findings, this suggests that the risk of C-PTSD from police work includes both repeated primary traumatic experiences (direct exposure to trauma) and exposure to vicarious trauma through others, such as the public and colleagues. Thus, future research in police officers must incorporate both primary and secondary trauma measures to accurately reflect the ways in which trauma symptoms develop in police officers.

The risk of C-PTSD in police officers with heightened anxiety and depression is consistent with evidence that anxiety and depression are commonly observed in those with C-PTSD [7]. Moreover, the relationship between fewer hours of sleep and C-PTSD is supported by evidence that poor sleep could be a risk factor for C-PTSD [8], although poorer sleep may also be a consequence of C-PTSD. However, the risk of PTSD and C-PTSD did not differ by the total frequency of traumatic events experienced personally and at work, inconsistent with evidence that chronic trauma is a significant risk factor for C-PTSD [2–4]. This indicates that in this sample of police officers clinical, lifestyle and occupational factors were more strongly associated with C-PTSD than personal trauma history. Given this, it is possible that trauma symptoms and the working environment interact to form a separate construct to PTSD and C-PTSD.

Longitudinal research in police officers has demonstrated that occupational factors are more predictive of PTSD symptoms at 12 months posttrauma than emotional and interpersonal factors [29]. Moreover, a systematic review of work-related PTSD in high-risk professionals, including police officers, established that PTSD following a work trauma can be mitigated through manager support, debriefing, and a solid organizational and psychosocial work environment [30]. Together with the present findings, this evidence supports the concept that occupational trauma has a specific development and distinct presentation of trauma symptoms. Hence, future research should improve the understanding of occupational trauma in police personnel.

This study has highlighted the possible importance of occupational factors in trauma symptoms in police officers. Potential modifiable factors were identified that could improve psychological well-being for police officers, for example good manager support and mediating work stress. This could be facilitated by developing awareness training of PTSD and C-PTSD and stress reduction programmes, focusing on supporting resilience among individuals and teams before and after traumatic work incidents. High-quality longitudinal research in all police officers, not just high-risk groups, is also necessary to confirm the generalizability of the risk factors. Finally, similar research should be conducted in other high-risk occupations, such as firefighters and paramedics, to clarify the mechanisms of occupational factors in C-PTSD. Further research will refine our understanding of trauma symptoms and C-PTSD presentation in police officers, and ultimately enhance outcomes for this population.

## Supplementary Material

kqab114_suppl_Supplementary_Material

kqab114_suppl_Supplementary_Tables_S3_S4
