# Supplementary material for: Risk factors for complex posttraumatic stress disorder in UK police
Source: Occup Med (Lond). 2021 Aug 20;71(8):351–7. doi: 10.1093/occmed/kqab114 (PMC8849141; doi:10.1093/occmed/kqab114)
Supplement: kqab114_suppl_Supplementary_Material [file kqab114_suppl_Supplementary_Material.docx]

**Supplementary Material**

**Table of Contents**

| **Content** | **Page Number** |
| --- | --- |
| Information about screening booklet | 2-5 |
| Consent statement | 6 |
| Data protection policy | 7-11 |
| Lifestyle questionnaire | 12 |
| Employment background questionnaire | 13 |
| Analysis of missing data | 14-17 |

| 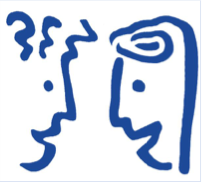  Why am I being asked to be psychologically |
| --- |
| screened? |
| Pre Completion Qs and As  What is psychological screening?  *Psychological screening is a way of assessing psychological health and wellbeing using reliable psychological questionnaires. All the questionnaires used in the screening have been thoroughly tested and shown to identify where people are having difficulties. The best approach to completing the questionnaires is to do them quickly without thinking too much about your responses. Although it is possible to cheat, there are ways that cheating can be picked up.*  Why am I being screened?  *It has been found that some roles carry a higher risk than other roles. All the roles in the police have been assessed and your role has been found to be in the high risk category. The organization has a duty to take care of your health and has chosen to use screening to pick up any signs that you may need additional help and support. Being screened is a benefit to you and to the organization as no one wants to become unwell as a* |
| *result of their work.*  How does it work?  *The process is very simple. When it is time for you to be screened you will receive an email with a link to the screening questionnaire. You can link up to the questionnaire on a laptop or using your mobile phone if you prefer. If you don’t finish the questionnaire at one sitting you can save what you have done and return when you have more time. The results are fed back to the analysis team who create the screening results. You will be told when you are booked to see the counsellor or psychologist for a feedback session.*  How long will it take?  *Typically, it takes around an hour to complete the initial questionnaire and around 20 minutes to complete the follow-up questionnaires. In some roles there is annual screening and in higher risk roles the screening can be every six months. In both cases there are usually face to face wellbeing sessions.*  What happens to the information?  *The information is encrypted and the whole system has been checked for any data protection issues. The information is sent to a confidential Occupational Health hub where it is stored. You can request copies of the report and also of the wellbeing report which is written following your face to face session. Management will get a fitness note together with any advice or offers of support. Where there needs to be counselling or a change of role there will be management advice. You will see any information that goes to management before it is sent.* |
| How does it help?  *We have found that most officers and staff who have been through the screening find it to be interesting and helpful. The process also provides management information which can highlight where there are problems in a team or where a organizational change has had a beneficial or detrimental impact. There have been lots of occasions where the screening programme has identified some underlying problem which has been resolved following the screening.* |

Who else is going through this process?

*The use of psychological screening is growing, many police forces are using this approach to protect the health and wellbeing of their officers and staff. In addition, a number of other emergency services are beginning to adopt the approach including fire fighters and paramedics. Recently a number of humanitarian organizations are looking to use screening with their workers.*

What will happen after I complete the online questionnaire?

*You will be asked to attend a session with a counsellor. During the session you will be asked about yourself so that the counsellor will get to know you better. The notes from the session are written up and you can ask for a copy of the report and also the fitness note that goes to your manager. If you need extra support you may be asked to speak to a psychologist who will be able to refer you for extra help should you need it.*

What happens if I am feeling stressed?

*If you are feeling stressed or burnt out when you complete the questionnaire, then this will show up in the*

*results. It is important that you are honest as the counsellor will see that you have needs and will be able to see from the results what can be done to help you recover.*

Why am I being asked about my childhood?

*In the initial screening there is a section which looks at childhood experiences. It has been found that early life events can have a big impact on physical, psychological and social health and wellbeing. If we know that you have had early difficulties, we can work with you to make sure that you get the best possible support so that your experiences can help you become more resilient rather than have problems.*

What kinds of symptoms should I notice?

*The symptoms that are measured by the questionnaire include anxiety, depression, burnout and trauma. There are also some subsets of symptoms in trauma these are:*

- *Avoidance which includes avoiding people, places or things associated with a trauma.*
- *Arousal which includes feeling jumpy, irritable and unable to sleep and*
- *Re-experience where you have dreams, flashbacks and constant thinking about an aspect of the*

*trauma.*

What happens if I need extra help?

*If the counsellor or psychologist thinks that you need extra help a recommendation will be made to refer you for trauma therapy or other appropriate treatment. Often all that is required is some advice and support to get you back to health.*

How can I become more resilient?

*Building coping skills, adopting a healthy lifestyle and feeling confident and capable in your role all help to make you resilient. Wherever possible we will recommend education programmes to help you build your wellbeing and resilience.*

What happens if I have problems between sessions?

*It is important that if you are noticing that you are not coping with the work you report this as soon as possible. You will be able to do this directly to the counselling service who will decide on the best way forward.*

**Consent Statement**

*I undertake to answer these questions honestly and consent to information from my screening being held securely on my Occupational Health File, where applicable.  I understand that anonymised screening data may be aggregated to provide management information, used in research and to improve services. I understand that the results of the screening are covered by Data Protection Legislation.*

**Data Protection Policy**

| Privacy Policy Noreen Tehrani Associates Psychological Screening | 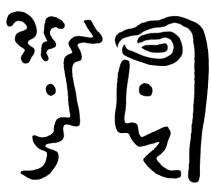 |
| --- | --- |

**Noreen Tehrani Associates Psychological Screening Ltd. (NTAPS)** are committed to protect and respect your privacy.

NTA Psychological Screening Ltd (registration number: 09869450) of Fourth Floor, 18-20 Hill Rise, Richmond, England, TW10 6UA is registered with the ICO (registration number: ZA169767). The ICO is the UK's independent body set up to uphold information rights. The ICO contact details are:
• https://ico.org.uk

- Information Commissioner's Office, Wycliffe House, Water Lane, Wilmslow, Cheshire, SK9 5AF
- 0303 123 1113

NTA Psychological Screening provides psychological screening questionnaires and associated psychological services.

By consenting to one of our questionnaires or engaging with one of our services, you agree to our Privacy Policy. Specifically, you expressly consent to us processing your personal data as described in this Privacy Policy & Notice. This Privacy Policy & Notice does not provide any additional terms and conditions nor warranties whether expressly or implied. This Privacy Policy & Notice provides transparency to our users as how their data is collected and used and serves as a privacy notice as required by legislation.

**NTAPS Screening:**

We issue an invitation to you to complete a psychological screening questionnaire on the instruction of the Data Controller. The Data Controller, usually your employer, will be indicated on the screening invite. We will not contact you other than in performance of the Data Controller’s instructions or to respond to a query from you.

The Data Controller’s instruction will include information about you to help us administer the screening such as your name, contact email address, your team (where appropriate) and purpose of the screening.

When you complete the screening questions, we store those question responses for the Data Controller. If you would like your questionnaire responses erased, rectified, accessed or for any other queries about your responses, please contact the Data Controller directly.

Tracking data will also be collected as you complete the questionnaire, such as opening the invitation email, logging into the questionnaire, times when the questionnaire was started and completed and the IP address of the device on which the questionnaire was completed.

**NTAPS Trauma Therapy:**

Trauma therapy is commenced with your consent. Screening results, assessments and management reports related to your trauma therapy will be held on your Confidential Medical File. Where your employer has an Occupational Health Department, this data will be held by them. You can request access to all screening and other reports written about you from your employer (Occupational

Health). Any management guidance produced to support my recovery/return to work will be agreed with you before it is sent to your manager/supervisor by Occupational Health.

You will be asked to complete questionnaires and provide feedback on the trauma therapy at the end of the sessions. Feedback and questionnaire results will be used to evaluate the programme and the interventions. That the purpose of any research or evaluations undertaken on your data will be used to improve the trauma support programme and the treatment of victims of trauma. Anonymised management information on the effectiveness of the programme will be provided to your employer/data controller. You can withdraw your data from any research (but not from the surveillance) without affecting your right to the trauma therapy

**NTAPS’ Use of the information:**

We use your questionnaire responses to carry out the instruction of the Data Controller. Based on the questionnaire responses we will produce a psychological screening report. Where the Data Controller (e.g. your employer) has an Occupational Health service provider, we will issue the report to them.

Data from the screening questionnaire may also be shared with a NTAPS Psychologist or Trauma Therapist to carry out further assessment or therapy as instructed by the Data Controller. Please refer to your Data Controller if you have any questions regarding their data privacy and data handling policies.

Questionnaire responses may be aggregated anonymously to provide management information to the Data Controller and to perform statistical research to improve our services.

**Your rights to the data we process on behalf of the Data Controller:**

If you want to access your data, rectify your data, erase your data, object to the processing of your data, or for any other enquiries regarding the handling of your data, please contact the Data Controller directly.

**Data Retention:**

The Data Controller retains control of the questionnaire data. Please refer to them regarding their policies on retention of data.

**Data Security:**

NTAPS employs generally accepted standards and best practice standards of security for information both during transmission and once we receive it. NTAPS has active security measures to ensure that we are compliant with all data we process in accordance to data privacy legislation as applicable to the UK.

NTAPS data security is designed to protect your personally identifiable information and your data from loss, misuse or unauthorized access, disclosure, alteration or destruction. Our technical and organisational security measures mean that we comply with our obligations to ensure that data stored with us is stored safely and securely.

Our security measures include • Access controls
• Firewalls

- Online Security (TLS encryption)
- Encryption in transit and at rest

If you have any questions, please contact support@noreentehrani.com for further details.

**Sharing and Disclosure of your data:**

We use sub-processors to collect and store your data safely and securely. All survey data is stored in the EEA. All our sub-processors have robust security features to ensure that they have the appropriate technical and organisation measures to keep personal data secure and backed up. If you have a query regarding our sub-processors, please contact: support@noreentehrani.com

**Right to complain:**

If you feel that we have mistreated the handling of your data, you have the right to complain to us in which case we will resolve the matter as quickly as possible to prevent any further risk of mishandling. You also have the right to complain to the Information Commissioner’s Office.

**Necessary Disclosure by law:**

Though we make every effort to preserve user privacy, we may need to disclose personal information when required by law wherein we have a good-faith belief that such action is necessary to comply with a current judicial proceeding, a court order or legal process served.

**Changes to this policy:**

We may change this Privacy Policy & Notice at any time by updating this document. Please review this document frequently.

**General definitions:**

Data Controller: the natural or legal person, public authority, agency or other body which, alone or jointly with others, determines the purposes and means of the processing of personal data; where the purposes and means of such processing are determined by Union or Member State law, the controller or the specific criteria for its nomination may be provided for by Union or Member State law.

Personal data: any information relating to an identified or identifiable natural person (“Data Subject”). An identifiable natural person is one who can be identified, directly or indirectly, in particular by reference to an identifier such as a name, an identification number, location data, an online identifier or to one or more factors specific to the physical, physiological, genetic, mental, economic, cultural or social identity of that natural person.

Data subject: any identified or identifiable natural person, whose personal data is processed by the controller responsible for the processing.

Processor: a natural or legal person, public authority, agency or other body which processes personal data on behalf of the controller.

Recipient: a natural or legal person, public authority, agency or another body, to which the personal data are disclosed, whether a third party or not. However, public authorities which may receive personal data in the framework of a particular inquiry in accordance with Union or Member State law shall not be regarded as recipients; the processing of those data by those public authorities shall be in compliance with the applicable data protection rules according to the purposes of the processing.

Third Party: a natural or legal person, public authority, agency or body other than the data subject, controller, processor and persons who, under the direct authority of the controller or processor, are authorised to process personal data.

Restriction of processing: the marking of stored personal data with the aim of limiting their processing in the future.

Processing: any operation or set of operations which is performed on personal data or on sets of personal data, whether or not by automated means, such as collection, recording, organisation, structuring, storage, adaptation or alteration, retrieval, consultation, use, disclosure by transmission, dissemination or otherwise making available, alignment or combination, restriction, erasure or destruction.

Profiling: any form of automated processing of personal data consisting of the use of personal data to evaluate certain personal aspects relating to a natural person, in particular to analyse or predict aspects concerning that natural person's performance at work, economic situation, health, personal preferences, interests, reliability, behaviour, location or movements.

Consent: Consent of the data subject is any freely given, specific, informed and unambiguous indication of the data subject's wishes by which he or she, by a statement or by a clear affirmative action, signifies agreement to the processing of personal data relating to him or her.

**Legal Basis:**

In addition to the legal basis mentioned above, we may also possess the following legal basis for processing the Data Subject’s personal data where:

- the data subject has given consent to the processing of his or her personal data for one or more specific purposes;
- processing is necessary for the performance of a contract to which the data subject is party or in order to take steps at the request of the data subject prior to entering into a contract;
- processing is necessary for compliance with a legal obligation to which the controller is subject;
- processing is necessary in order to protect the vital interests of the data subject or of another natural person;
- processing is necessary for the performance of a task carried out in the public interest or in the exercise of official authority vested in the controller; or,
- processing is necessary for the purposes of the legitimate interests pursued by the controller or by a third party, except where such interests are overridden by the interests or fundamental rights and freedoms of the data subject which require protection of personal data, in particular where the data subject is a child.

**Law & jurisdiction:**

This Privacy Policy & Notice is governed by and interpreted according to the law of England and Wales. All disputes arising out of this Privacy Policy & Notice will be subject to the exclusive jurisdiction of the English and Welsh courts.

**Transfer of Rights:**

You may not transfer any of your rights under this Privacy Policy & Notice to any other person. We may transfer our rights under this privacy notice where we reasonably believe your rights will not be affected.

**Invalid provisions:**

If any court or competent authority finds that any provision of this Privacy Policy & Notice (or part of any provision) is invalid, illegal or unenforceable, that provision or part-provision will, to the extent required, be deemed to be deleted, and the validity and enforceability of the other provisions of this Privacy Policy & Notice will not be affected.

**Data Subject Rights:**

Certain regulations afford EU Data Subjects with rights. These rights are listed below. In order to assert any of these rights please contact your Data Controller. Any Data Subject Requests that NTAPS receive will be forwarded to the Data Controller. More information on these rights can be found on the ICO website.

- The right to be informed
- The right of access
- The right of rectification
- The right of erasure
- The right to restrict processing
- The right to data portability
- The right to object
- Rights in relation to automated decision making and profiling

13.09.2018

**Lifestyle Questionnaire**

Scores are given as either ‘Good’, ‘OK’, or ‘Could be improved’. For each questions, the first responses are scored ‘Good’, second responses ‘OK’ and final responses ‘Could be improved’.

- On average, how many meals do you have per day?
- *3 or more*
- *2*
- *1*
- On average, how many caffeine drinks do you have per day?
- *Less than 4*
- *Between 5 and 6*
- *6 or more*
- On average, how many units of alcohol do you drink per week?
- *8 or less*
- *Between 8 and 14*
- *More than 14*
- On average, how many times do you exercise outside of work per week?
- *More than 3 times*
- *Between 1 and 3 times*
- *Not at all*
- How many interests or hobbies do you have outside of work?
- *More than 2*
- *1 or 2*
- *None*
- On average, how many times do you meet with friends outside of work per week?
- *More than 2 times*
- *1 or 2 times*
- *Not at all*
- On average, how many hours of sleep do you get per night?
- *More than 6*
- *Between 5 and 6*
- *Less than 5*
- On average, how many cigarettes do you smoke per day?
- *None*
- *Between 1 and 10*
- *More than 10*

**Employment Background Questionnaire**

- How much exposure do you have to traumatic events, materials or information?

*(High/Medium/Low/None)*

- What, if any, motivation do you have to leave your current role?

*(High/Medium/Low/None)*

- In general, what would you say is the state of your health?

*(Excellent/Very Good/Good/Fair/Poor)*

- People’s personal ability to work varies over their working life. How would you compare your current capability to work, compared to your lifetime best?

*(Excellent/Very Good/Good/Fair/Poor)*

- How stressful do you find your job?

*(Extremely/Very/Moderately/Mildly/Not at all)*

- How do you find the support you receive from your line manager?

*(Excellent/Very Good/Good/Fair/Poor)*

**Analysis of Missing Data**

Data is only missing on the 6 ‘Employment Background’ exposure variables. The percentage of missing data on these variables can be found in Supplementary Table 1.

To examine predictors of missingness, a binary variable was generated indicating ‘missing’ vs ‘no missing’ data for each participant based on whether the participant had missing data on either one of the 6 ‘Employment Background’ exposure variables. Logistic regression analyses were performed with this ‘missing’ vs ‘non missing’ binary variable as the outcome and each variable as the exposures to examine predictors of missingness (Table S2). Variables with a strong association with missing data were considered for adjustment in the multivariable logistic regression models. Variables were selected for adjustment if they were thought to be confounders and not on the causal pathway, so as not to adjust for any mediators of the association between the exposure variables and the outcome of PTSD or C-PTSD.

Due to associations with missingness, age, tenure and job group were controlled for in each model.

*Table S1:* Total number and percentage of missing data on each variable.

|  | **Total Police Officers *(n* = 2,444)** |
| --- | --- |
| **Variable** | ***n* (%) of missing data** |
| Perceived exposure to traumatic material at work | 470 (19%) |
| Intentions to leave role | 470 (19%) |
| Health beliefs | 470 (19%) |
| Work stress | 470 (19%) |
| Perceived manager support | 470 (19%) |
| Workability | 499 (20%) |

*Table S2:* Logistic regression analyses between variables and missing vs not missing data variable.

|  | **Odds Ratio** | **95% Confidence Interval** | ***p-*value** |
| --- | --- | --- | --- |
| **Age (Years)** | 0.94 | 0.93 to 0.95 | <0.001 |
| **Gender (Male)** | 0.93 | 0.77 to 1.13 | 0.483 |
| **Screening Type  (Routine)** | -^†^ |  |  |
| **Positive Screening for PTSD** | 0.25 | 0.11 to 0.56 | 0.001 |
| **Positive Screening for C-PTSD** | 0.07 | 0.02 to 0.21 | <0.001 |
| **Anxiety** | 0.70 | 0.67 to 0.74 | <0.001 |
| **Depression** | 0.65 | 0.61 to 0.70 | <0.001 |
| **Personal History (*n*)** |  |  |  |
| Adverse childhood events | 0.87 | 0.80 to 0.94 | 0.001 |
| Adverse adult events | 0.89 | 0.84 to 0.95 | 0.001 |
| Recent events | 0.73 | 0.63 to 0.84 | <0.001 |
| Addictive behaviours | 0.77 | 0.60 to 1.00 | 0.046 |
| Total | 0.91 | 0.87 to 0.94 | <0.001 |
| **Emotional Awareness** |  |  |  |
| Dissociation | 1.31 | 1.23 to 1.40 | <0.001 |
| Physical sensitivity | 0.80 | 0.74 to 0.86 | <0.001 |
| Emotional sensitivity | 0.97 | 0.91 to 1.03 | 0.350 |
| Sensory awareness | 1.36 | 1.27 to 1.47 | <0.001 |
| Empathy | 1.20 | 1.13 to 1.27 | <0.001 |
| Interpersonal sensitivity | 1.58 | 1.44 to 1.73 | <0.001 |
| Emotional resilience | 1.77 | 1.57 to 1.99 | <0.001 |
| **Alcohol (units per week)** |  |  |  |
| 7 or less | 1.00 | - | - |
| 8-14 | 0.54 | 0.39 to 0.74 | <0.001 |
| 15 or more | 0.45 | 0.28 to 0.70 | 0.001 |
| **Socialising Outside of Work (*n* per week)** |  |  |  |
| 3 times or more | 1.00 | - | - |
| 1-2 times | 0.58 | 0.44 to 0.75 | <0.001 |
| None | 0.21 | 0.15 to 0.31 | <0.001 |
| **Sleep (hours per night)** |  |  |  |
| 7 or more | 1.00 | - | - |
| 5-6 | 0.35 | 0.27 to 0.44 | <0.001 |
| Less than 5 | 0.15 | 0.06 to 0.36 | <0.001 |
| **Professional Quality of Life** |  |  |  |
| Compassion satisfaction | 1.10 | 1.09 to 1.12 | <0.001 |
| Burnout | 0.92 | 0.90 to 0.93 | <0.001 |
| Compassion fatigue | 0.90 | 0.88 to 0.92 | <0.001 |
| **Sense of Coherence** |  |  |  |
| Meaningfulness | 1.14 | 1.11 to 1.17 | <0.001 |
| Comprehensibility | 1.11 | 1.09 to 1.13 | <0.001 |
| Manageability | 1.13 | 1.10 to 1.15 | <0.001 |
| **Tenure** |  |  |  |
| Not in role | 1.00 | - | - |
| 0-6 months | 0.02 | 0.01 to 0.04 | <0.001 |
| 7-12 months | 0.00 | 0.00 to 0.01 | <0.001 |
| 13-18 months | 0.01 | 0.01 to 0.02 | <0.001 |
| 19-24 months | 0.01 | 0.01 to 0.02 | <0.001 |
| 2-3 years | 0.02 | 0.01 to 0.03 | <0.001 |
| 3-4 years | 0.01 | 0.01 to 0.03 | <0.001 |
| 4-5 years | 0.04 | 0.02 to 0.07 | <0.001 |
| 5-6 years | 0.03 | 0.01 to 0.05 | <0.001 |
| >6 years | 0.01 | 0.01 to 0.02 | <0.001 |
| **Job Group** |  |  |  |
| Investigations | 1.00 | - | - |
| Community policing | 2.10 | 1.54 to 2.86 | <0.001 |
| Intelligence | 1.01 | 0.56 to 1.82 | 0.971 |
| Operational support | 1.14 | 0.83 to 1.55 | 0.414 |
| Unknown | 4.10 | 3.19 to 5.28 | <0.001 |
| **Perceived Exposure to Traumatic Material at Work** |  |  |  |
| None/Low | 1.00 | - | - |
| Moderate | 0.49 | 0.21 to 1.13 | 0.095 |
| High | 0.30 | 0.11 to 0.81 | 0.017 |
| **Intentions to Leave Role** |  |  |  |
| None | 1.00 | - | - |
| Low | 1.51 | 0.65 to 3.53 | 0.337 |
| Moderate | 0.88 | 0.12 to 2.44 | 0.804 |
| High | 0.22 | 0.03 to 1.75 | 0.153 |
| **Health Beliefs** |  |  |  |
| Poor/Fair | 1.00 | - | - |
| Good | 5.00 | 0.63 to 39.64 | 0.127 |
| Excellent | 8.58 | 1.14 to 64.34 | 0.036 |
| **Workability** |  |  |  |
| Poor/Fair | -* | - | - |
| Good | - | - | - |
| Excellent | - | - | - |
| **Job Stress** |  |  |  |
| None/Mild | 1.00 | - | - |
| Moderate | 0.32 | 0.14 to 0.70 | 0.004 |
| High | 0.10 | 0.02 to 0.44 | 0.002 |
| **Perceived Manager Support** |  |  |  |
| Poor/Fair | 1.00 | - | - |
| Good | 2.20 | 0.26 to 18.99 | 0.473 |
| Very Good | 2.29 | 0.29 to 17.87 | 0.429 |
| Excellent | 3.56 | 0.46 to 27.58 | 0.224 |

*Note:* ^†^categories too small to calculate estimate. *outcome does not vary.
