# Supplementary material for: Risk factors for complex posttraumatic stress disorder in UK police
Source: Occup Med (Lond). 2021 Aug 20;71(8):351–7. doi: 10.1093/occmed/kqab114 (PMC8849141; doi:10.1093/occmed/kqab114)
Supplement: kqab114_suppl_Supplementary_Tables_S3_S4 [file kqab114_suppl_Supplementary_Tables_S3_S4.pdf]

Table S3: Logistic regression analyses between occupational characteristics and positive screenings for PTSD and C-PTSD, unadjusted and adjusted for age, gender, tenure and job group.

	**Positive Screenings for PTSD (*n* = 98)**					**Positive Screenings for C-PTSD (*n* = 165)**				
		**Unadjusted**		**Adjusted**			**Unadjusted**		**Adjusted**	
	***M (SD) or n* (%)**	**OR**	**95% CI**	**OR**	**95% CI**	***M (SD) or n* (%)**	**OR**	**95% CI**	**OR**	**95% CI**
**Professional quality of life (ProQOL)**										
Compassion Satisfaction	32.6 (9.7)	0.96***	0.94 to 0.98	0.98	0.96 to 1.00	25.2 (13.4)	0.90***	0.89 to 0.91	0.92***	0.91 to 0.94
Burnout	26.9 (6.4)	1.06**	1.02 to 1.09	1.03	0.99 to 1.07	28.1 (7.0)	1.10***	1.07 to 1.13	1.06***	1.03 to 1.10
Compassion Fatigue	21.3 (11.1)	1.08***	1.06 to 1.09	1.07***	1.05 to 1.09	30.2 (11.4)	1.17***	1.15 to 1.19	1.15***	1.12 to 1.17
**Sense of Coherence (SoC)**										
Meaningfulness	18.6 (5.4)	0.89***	0.86 to 0.92	0.92***	0.89 to 0.95	14.8 (5.5)	0.79***	0.76 to 0.81	0.81***	0.78 to 0.84
Comprehensibility	21.6 (5.1)	0.91***	0.88 to 0.93	0.94***	0.91 to 0.97	15.4 (5.9)	0.77***	0.75 to 0.79	0.80***	0.77 to 0.82
Manageability	15.4 (5.0)	0.88***	0.85 to 0.91	0.91***	0.88 to 0.94	10.8 (4.6)	0.73***	0.70 to 0.76	0.76***	0.73 to 0.79
**Area of work**										
Investigations	50 (51%)	1.00	-	1.00	-	52 (32%)	1.00	-	1.00	-
Community Policing	7 (7%)	0.60	0.27 to 1.34	0.67	0.30 to 1.51	13 (8%)	1.10	0.59 to 2.04	1.09	0.58 to 2.05
Intelligence	1 (1%)	0.25	0.03 to 1.82	0.27	0.04 to 1.98	1 (1%)	0.24	0.03 to 1.75	0.28	0.04 to 2.09
Operational Support	5 (5%)	0.30*	0.12 to 0.76	0.32*	0.13 to 0.82	2 (1%)	0.11**	0.03 to 0.47	0.11**	0.03 to 0.44
Unknown	35 (36%)	2.20**	1.40 to 3.43	2.88***	1.80 to 4.60	97 (59%)	6.98***	4.87 to 9.99	8.67***	5.91 to 12.72
**Tenure (years)**										
Recruitment/pre-deployment	1 (1%)	1.00	-	1.00	-	3 (2%)	1.00	-	1.00	-
0-6 months	14 (14%)	11.76*	1.54 to 90.01	17.83**	2.30 to 138.21	11 (7%)	3.03	0.84 to 10.97	6.80**	1.83 to 25.21
7-12 months	14 (14%)	17.29**	2.26 to 132.44	27.40**	3.53 to 212.80	11 (7%)	4.44*	1.22 to 16.09	10.75***	2.88 to 40.15
13-18 months	9 (9%)	12.78*	1.61 to 101.67	18.58**	2.31 to 149.73	12 (7%)	5.73**	1.59 to 20.56	12.95***	3.50 to 47.95
19-24 months	11(11%)	22.0**	2.81 to 172.09	31.42**	3.96 to 249.12	16 (10%)	10.98***	3.15 to 38.31	22.63***	
2-3 years	8 (8%)	10.33*	1.28 to 83.19	15.08*	1.85 to 122.61	13 (8%)	5.68**	1.60 to 20.20	11.62***	3.18 to 42.40
3-4 years	10 (10%)	18.44**	2.34 to 145.41	26.55**	3.33 to 211.44	17 (10%)	10.87***	3.13 to 37.70	20.26***	5.65 to72.65
4-5 years	1 (1%)	2.12	0.13 to 34.14	3.04	0.19 to 49.35	11 (7%)	8.34**	2.29 to 30.41	16.75***	4.44 to 63.21
5-6 years	3 (3%)	10.14*	1.04 to 98.79	13.72*	1.39 to 135.00	5 (3%)	5.73*	1.34 to 24.49	9.74**	2.19 to 43.29
>6 years	27 (28%)	13.69*	1.85 to 101.28	18.79**	2.51 to 140.62	66 (40%)	11.91***	3.71 to 38.22	18.96***	5.79 to 62.09
**Perceived exposure to traumatic material at work**										
None/Low	14 (15%)	1.00	-	1.00	-	23 (14%)	1.00	-	1.00	-
Moderate	36 (39%)	1.06	0.56 to 1.99	1.16	0.61 to 2.22	50 (31%)	0.86	0.53 to 1.48	0.99	0.56 to 1.74
High	43 (46%)	1.38	0.75 to 2.56	1.37	0.72 to 2.58	90 (55%)	1.83*	1.14 to 2.95	1.59	0.93 to 2.71
**Intentions to leave role**										
None	.83 (26%)	1.00	-	1.00	-	33 (21%)	1.00	-	1.00	-
Low	29 (31%)	1.54	0.89 to 2.68	1.32	0.75 to 2.34	39 (24%)	1.47	0.91 to 2.36	1.18	0.70 to 1.99
Medium	22 (24%)	1.36	0.75 to 2.46	1.10	0.59 to 2.02	46 (28%)	2.09*	1.32 to 3.32	1.56	0.93 to 2.60
High	18 (19%)	1.73	0.92 to 3.24	1.00	0.97 to 1.02	44 (27%)	3.24***	2.02 to 5.19	1.94*	1.14 to 3.30
**Health beliefs**										
Poor/Fair	25 (27%)	1.00	-	1.00	-	91 (56%)	1.00	-	1.00	-
Good	45 (48%)	0.99	0.60 to 1.64	1.45	0.85 to 2.48	44 (27%)	0.22***	0.15 to 0.32	0.35***	0.23 to 0.53

For Peer Review

Table S3: Logistic regression analyses between occupational characteristics and positive screenings for PTSD and C-PTSD, unadjusted and adjusted for age, gender, tenure and job group.

Excellent	23 (25%)	0.39**	0.22 to 0.70	0.70	0.38 to 1.31	28 (17%)	0.11***	0.07 to 0.17	0.25***	0.15 to 0.40
**Workability**										
Poor/Fair	50 (54%)	1.00	-	1.00	-	119 (73%)	1.00	-	1.00	-
Good	30 (33%)	0.48**	0.30 to 0.76	0.69	0.41 to 1.17	35 (22%)	0.20***	0.14 to 0.30	0.41***	0.26 to 0.63
Excellent	12 (13%)	0.14***	0.07 to 0.26	0.21***	0.11 to 0.42	8 (5%)	0.03***	0.02 to 0.07	0.08***	0.04 to 0.18
**Work stress**										
None/Mild	16 (17%)	1.00	-	1.00	-	19 (12%)	1.00	-	1.00	-
Moderate	45 (48%)	1.58	0.88 to 2.82	1.57	0.87 to 2.84	49 (30%)	1.45	0.84 to 2.48	1.64	0.92 to 2.93
High	32 (34%)	1.82	0.99 to 3.36	1.37	0.73 to 2.60	95 (58%)	5.14***	3.09 to 8.55	3.91***	2.24 to 6.84
**Perceived manager support**										
Poor/Fair	7 (7%)	1.00	-	1.00	-	47 (29%)	1.00	-	1.00	-
Good	24 (26%)	1.53	0.65 to 3.63	2.38	0.98 to 5.82	36 (22%)	0.27***	0.17 to 0.43	0.35***	0.20 to 0.60
Very Good	33 (35%)	0.97	0.42 to 2.24	1.55	0.65 to 3.67	46 (28%)	0.16***	0.10 to 0.24	0.23***	0.14 to 0.38
Excellent	29 (31%)	1.22	0.53 to 2.84	1.86	0.78 to 4.46	34 (21%)	0.16***	0.10 to 0.26	0.23***	0.13 to 0.40

Note: *p<0.05, **p<0.01, ***p<0.001. †categories too small to calculate estimate. n = total number. M = mean. SD = standard deviation. OR = odds ratio. 95% CI = 95% confidence interval. p = p-value. ProQOL = Professional Quality of Life, a 30-item measure of the positive and negative consequences of working with others who have experienced stressful events, with possible subscale scores ranging from 10 to 50. SoC = Sense of Coherence, a 13-item measure of personal resilience with scores ranging from 13-91. The amount and percentage of missing data is as followed: Trauma at work (PTSD) = 5 (5%), trauma at work (CPTSD) = 2 (1%); Intentions to leave (PTSD) = 5 (5%), intentions to leave (CPTSD) = 2 (1%); Health beliefs (PTSD) = 5 (5%), health beliefs (CPTSD) = 2 (1%); Workability (PTSD) = 6 (6%), workability (CPTSD) = 3 (2%); Job stress (PTSD) = 5 (5%), job stress (CPTSD) = 2 (1%); Manager support (PTSD) = 5 (5%), manager support (CPTSD) = 2 (1%).

For Peer Review

Table S4: Logistic regression analyses between demographic, clinical and lifestyle characteristics and positive screenings for PTSD and C-PTSD, unadjusted and adjusted for age, gender, tenure and job group.

	**Positive Screenings for PTSD (*n* = 98)**					**Positive Screenings for C-PTSD (*n* = 165)**				
		**Unadjusted**		**Adjusted**			**Unadjusted**		**Adjusted**	
	***M* (SD) or *n* (%)**	**OR**	**95% CI**	**OR**	**95% CI**	***M* (SD)* or *n* (%)**	**OR**	**95% CI**	**OR**	**95% CI**
**Age (years)**	39.8 (8.8)	1.00	0.98 to 1.02	1.00	0.98 to 1.02	41.9 (8.6)	1.03**	1.01 to 1.05	1.03**	1.01 to 1.05
**Gender (male)**	43 (44%)	0.69	0.46 to 1.04	0.70	0.46 to 1.07	85 (52%)	0.95	0.69 to 1.30	0.82	0.58 to 1.17
**Anxiety (Goldberg)**	7 (5-8)	1.45***	1.34 to 1.57	1.39***	1.27 to 1.52	8 (7-9)	2.07***	1.87 to 2.30	1.88***	1.68 to 2.09
**Depression (Goldberg)**	5 (3-7)	1.38***	1.29 to 1.48	1.29***	1.19 to 1.40	7 (7-8)	2.27***	2.04 to 2.53	2.08***	1.86 to 2.33
**Personal History**										
Childhood Events	0 (0-2)	1.23***	1.11 to 1.36	1.15**	1.04 to 1.28	1 (0-2)	1.30***	1.20 to 1.41	1.22***	1.11 to 1.33
Adult Events	3 (1-4)	1.39***	1.25 to 1.54	1.27***	1.14 to 1.43	4 (1-5)	1.79***	1.64 to 1.96	1.58***	1.43 to 1.74
Recent Events	1 (0-1)	1.42***	1.15 to 1.75	1.28*	1.03 to 1.58	1 (0-1)	1.54***	1.31 to 1.82	1.37**	1.15 to 1.65
Addictive Behaviours	0 (0-0)	1.43*	1.00 to 2.04	1.21	0.84 to 1.73	0 (0-1)	2.46***	1.95 to 3.11	2.14***	1.64 to 2.80
Total	4 (2-7)	1.18***	1.12 to 1.25	1.13***	1.06 to 1.19	6 (3-9)	1.31***	1.25 to 1.37	1.23***	1.17 to 1.28
**Emotional Awareness (ELQ)**										
Dissociation	2.5 (2.4)	0.70***	0.64 to 0.76	0.75***	0.66 to 0.85	1.4 (2.2)	0.51***	0.47 to 0.56	0.61***	0.55 to 0.69
Physical Sensitivity	2.0 (2.1)	1.27***	1.14 to 1.42	1.37***	1.21 to 1.55	1.2 (2.0)	0.95	0.85 to 1.05	1.18**	1.05 to 1.33
Emotional Sensitivity	2.0 (2.1)	1.14*	1.02 to 1.29	1.25***	1.11 to 1.41	1.2 (2.0)	0.81***	0.72 to 0.90	1.05	0.93 to 1.18
Sensory Awareness	3.0 (2.5)	0.74***	0.68 to 0.81	0.83**	0.73 to 0.95	1.7 (2.5)	0.53***	0.49 to 0.58	0.65***	0.58 to 0.72
Empathy	2.4 (2.2)	0.83**	0.74 to 0.94	0.97	0.86 to 1.11	1.3 (2.1)	0.52***	0.46 to 0.58	0.73***	0.65 to 0.83
Interpersonal Sensitivity	2.9 (2.6)	0.72***	0.66 to 0.78	0.76***	0.67 to 0.86	1.6 (2.4)	0.53***	0.49 to 0.57	0.61***	0.55 to 0.68
Emotional Resilience	2.9 (2.5)	0.71***	0.65 to 0.76	0.72***	0.64 to 0.81	1.4 (2.2)	0.52***	0.48 to 0.56	0.57***	0.51 to 0.63
**Alcohol units (*n* per week)**										
7 or less	73 (74%)	1.00	-	1.00	-	113 (68%)	1.00	-	1.00	-
8-14	13 (13%)	0.93	0.51 to 1.70	0.95	0.51 to 1.77	22 (13%)	1.02	0.64 to 1.64	0.98	0.58 to 1.64
15 or more	12 (12%)	1.67	0.89 to 3.14	1.45	0.74 to 2.86	30 (18%)	2.93***	1.90 to 4.53	2.17**	1.30 to 3.62
**Socialising outside of work (*n* per week)**										
3 or more	4 (4%)	1.00	-	1.00	-	15 (9%)	1.00	-	1.00	-
1-2 times	59 (60%)	2.81*	1.01 to 7.80	2.80	1.00 to 7.86	68 (41%)	0.84	0.47 to 1.49	0.71	0.39 to 1.32
0 times	35 (36%)	5.28**	1.86 to 15.01	4.28**	1.47 to 12.41	82 (50%)	3.51***	1.99 to 6.21	2.41**	1.29 to 4.50
**Sleep (hours per night)**										
7 or more	40 (41%)	1.00	-	1.00	-	37 (22%)	1.00	-	1.00	-
5-6	49 (50%)	2.32***	1.51 to 3.55	1.93*	1.24 to 2.99	87 (53%)	4.69***	3.16 to 6.95	3.81***	2.51 to 5.79
Less than 5	9 (9%)	3.69**	1.74 to 7.84	1.99	0.90 to 4.41	41 (25%)	23.26***	16.87 to 47.34	13.40***	7.52 to 23.89

Note:*p<0.05, **p<0.01, ***p<0.001. *n* = total number. *M* = mean. SD = standard deviation. OR = odds ratio. 95% CI = 95% confidence interval. p = p-value.
Goldberg = Goldberg Anxiety/Depression Scale, an 18-item inventory measuring symptoms of anxiety and depression with possible subscale scores ranging from 0 to 9. Emotional Awareness = Emotional Awareness Questionnaire, a 42-item scale measuring how someone recognises, translates and responds to their emotions with possible subscale scores ranging from 0 to 6.

For Peer Review
